# A Treatise on the Materia Medica and Therapeutics of the Skin

**Published:** 1881-03

**Authors:** 


A Treatise on the Materia Medica and Therapeutics of the Skin. By Henry G. Piffard, A.M., M.D., Professor of Dermatology, Medical Department of the City of New York. Wm. Wood & Co., New York. 1881.

